# Evidence of Metallic and Polyether Ionophores as Potent Therapeutic Drug Candidate in Cancer Management

**DOI:** 10.3390/molecules27154708

**Published:** 2022-07-23

**Authors:** Pratibha Pandey, Fahad Khan, Huda A. Qari, Tarun Kumar Upadhyay, Abdulhameed F. Alkhateeb, Mohammad Oves

**Affiliations:** 1 Department of Biotechnology, Noida Institute of Engineering and Technology, Greater Noida 201306, India; pratibhapandey.bio@niet.co.in; 2 Department of Biological Science, Faculty of Sciences, King Abdulaziz University, Jeddah 21589, Saudi Arabia; hagari@kau.edu.sa; 3 Department of Biotechnology, Parul Institute of Applied Sciences and Animal Cell Culture and Immunobiochemistry Lab, Centre of Research for Development, Parul University, Vadodara 391760, India; tarun_bioinfo@yahoo.co.in; 4 Department of Electrical & Computer Engineering, Faculty of Engineering, King Abdulaziz University, Jeddah 21589, Saudi Arabia; afalkhateeb@kau.edu.sa; 5 Center of Excellence in Environmental Studies, King Abdulaziz University, Jeddah 21589, Saudi Arabia; 6 Institute of Multidisciplinary Research for Advanced Materials (IMRAM), Tohoku University, Sendai 980-8577, Japan

**Keywords:** drug repurposing, ionophores, cancer, ion homeostasis, cell signaling pathways

## Abstract

Cancer remains one of the most crucial human malignancies with a higher mortality rate globally, and is predicted to escalate soon. Dysregulated ion homeostasis in cancerous cells prompted the researchers to investigate further ion homeostasis impeding agents as potent anticancerous agents. Reutilization of FDA-approved non-cancerous drugs has emerged as a practical approach to developing potent, cost-effective drugs for cancer treatment. Across the globe, most nations are incapable of fulfilling the medical demands of cancer patients due to costlier cancerous drugs. Therefore, we have inclined our review towards emphasizing recent advancements in cancer therapies involving ionophores utilization in exploring potent anticancer drugs. Numerous research reports have established the significant anticancerous potential of ionophores in several pre-clinical reports via modulating aberrant cell signaling pathways and enhancing antitumor immunity in immune cells. This review has mainly summarized the most significant ion homeostasis impeding agents, including copper, zinc, calcium, and polyether, that presented remarkable potential in cancer therapeutics via enhanced antitumor immunity and apoptosis induction. Altogether, this study could provide a robust future perspective for developing cost-effective anticancerous drugs rapidly and cost-effectively, thereby combating the limitations of currently available drugs used in cancer treatment.

## 1. Introduction

Enormous evidence strongly indicates the significance of altered ion transport in cancer progression [1]. Cancerous cells control their growth and proliferation by maintaining their ion pumps, ion homeostasis, and ion channels which are too crucial for their growth and survival [2]. Controlled regulation of the maintenance of ions in the extracellular matrix and inside the cells is vital for proper cell shape, maintained membrane potential, and normal cell signaling pathways. The structural organization of the plasma membrane leads to the impermeability of ions, macromolecules (e.g., RNA), and several other hydrophilic molecules (e.g., glucose) across the cellular membrane [3]. However, cells overrule these transport phenomena by developing several mechanisms for active transport and facilitated diffusion of molecules and ions across membranes. Facilitated transport constitutes ion diffusion towards a protein-mediated concentration gradient that leads to the formation of water-filled ion channels across the cell membrane. The gating of ion channels is controlled (opened or closed) depending on cellular requirements. Commonly reported gated ion channels include voltage-gated, ligand-gated, light-gated, and mechanically gated [4,5,6].

During normal cellular functioning, ions (or molecules) are transported using numerous active transporter proteins (H^+^/K^+^ ATPase, ABC transporters Ca^2+^ ATPase, ATP. Na^+^/K^+^ ATPase) against the concentration gradient [7,8,9]. Aberrant functioning (expression) of these ion channels and pumps in cancerous cells helps them regulate distinctive ion homeostasis that further helps them in growth and proliferation [10,11]. Ionophores (calcium, copper, zinc, and polyether) have widely been utilized in cancer treatment as chemo-sensitizers for maintaining the ion balance in cancerous cells [12,13,14]. Therefore, we have accumulated research evidence explaining how cancer cells utilize ion homeostasis for their growth and providing a potential cancer therapeutic approach. Further sections will uncover the anti-cancerous potential of numerous potent metal ionophores such as copper, calcium, and zinc.

## 2. Repurposing of Copper Ionophores in Cancer

Bioactive compounds, including flavones (O, O donor ligands), have been classified as copper ionophores with significant anticancerous potential. Numerous copper (Cu) ionophores have displayed a wider range of anticancerous potential either by ROS-mediated cytotoxicity or targeting specific signaling pathways [13,14,15,16]. Copper is preferentially transported to cuproenzymes in mitochondria, such as the antioxidant enzyme superoxide dismutase 1 and the respiratory chain complex IV (COX) (SOD1). The cuproenzyme COX participates in the mitochondrial respiratory chain by catalyzing the reduction of molecular oxygen to water. Copper plays a significant role in COX-mediated ATP synthesis, indicating that this bio-metal center is essential for maintaining essential functions. Important copper/zinc-containing antioxidant enzyme SOD1 (superoxide dismutase 1) is primarily found in the cytosol and in the intermembrane space of mitochondria. Furthermore, intracellular copper alters the mitochondrial function or triggers metabolic reprogramming to affect cancer cell proliferation or differentiation.

Copper (Cu) ionophores can be grouped into some significant classes, such as thiosemicarbazones (C_10_H_12_N_4_OS) (TSCs) and dithiocarbamates (DTCs), hydroxyquinolines (C_9_H_7_NO_4_S) (HQs), and hydroxyflavones (HFs) [17,18]. It is equally important to study the copper complexes’ physicochemical potential to determine the copper ionophores’ efficacy. Pyrrolidine dithiocarbamate (C_5_H_9_NS_2_) (PDTC) was the first identified Cu ionophores that showed its toxic efficacies via transport of redox-active copper into the cell that further inhibited cell proliferation, ubiquitin-proteasome/nuclear factor-jB mediated apoptosis induction in prostate and breast cancerous cells [19,20]. DTCs have exhibited significant metal uptake and apoptosis induction in numerous cancer cells. Disulfiram (C_10_H_20_N_2_S_4_) (DSF) is highly effective against various carcinomas such as prostate and breast carcinoma via targeting stem-like cancer cells and potentiating the cytotoxic efficacies of a broader range of anticancer drugs. However, phase II clinical trials of DSF in prostate cancer patients failed to produce significant benefits. Thus, DSF could only affect tumor growth of castrate-resistant and hormone-sensitive prostate cancer (xenografts) when co-administered with copper [21].

Numerous research studies reported ROS augmentation in cells treated with Cu and DSF, mainly due to the redox cycling of CuDTC (copper dithiocarbamates) complexes [22,23]. Cu-DSF (copper disulfiram) inhibited NF-kB (Nuclear factor-kappa B) activation and down-regulated XIAP (apoptotic protein and chemoresistance factor), thereby leading to pro-apoptotic response activation [24]. Cu-DSF drug not only triggered ROS but simultaneously inhibited NF-jB up-regulation resulting in potent improvements in ROS-targeted chemotherapeutic approaches [25]. A possible mechanism behind these improvements could be the involvement of copper ion since Cu-XIAP (copper X-linked inhibitor of apoptosis protein) complex formation induce apoptosis induction by inhibiting the anti-apoptotic potential of XIAP [26]. Cu-DSF also inhibited ALDH1 (Aldehyde Dehydrogenase 1), a potent anti-cancer target that can potentially sensitize cancer stem cells to traditional chemotherapeutic cancer drugs. Other targets of Cu-DSF have also been unrevealed, such as the p-97-NPL4-UFDI protein complex that mainly regulates proteasomes upstream, and its inhibition might inhibit proteasomes leading to ubiquitinated proteins accumulation [27].

Co-administration of Cu and DSF is selectively toxic to several malignant cells compared to healthy prostate cells. This property could be attributed to the ability of prostate cancer cells to accumulate Cu and the oxidative environment inside the cancerous cells, making them susceptible to ROS (reactive oxygen species). Further research displayed that Cu ionophores such as DSF could selectively target prostate cancer cells by releasing intra-cellularly Cu leading to ROS formation in TRAMP (transgenic adenocarcinoma of the mouse prostate) adenocarcinoma cells (TRAMP-C1). TRAMP-C1 cells have a lower ability to protect from oxidative stress [28]. Cu-DSF treatment reduced GSH levels in prostate cancer cells and increased intracellular GSH (reduced form of glutathione) in normal prostate epithelial cells [29]. Co-administration of Cu and 2, 20-dithiodipyridine (DPy) is highly cytotoxic against various cancer cells, including HEPG2 (human liver cancer cell line), SMMC-7721 (human hepatoma cell line), HeLa (cervical cancer cells), and A549 (human lung cancer cells) cancer cells via enhancing intracellular Cu levels in a time-dependent manner in comparison to their effects when either of them is administered alone [30]. Moreover, their synergistic efficacies (DPy and Cu) have not been reported on normal cells such as BEAS-2B (epithelial cell line), L02 (cellosaurus cell line), and HEK 293 (human embryonic kidney) T cells [31].

After that, the research shifted towards developing novel bis (thiosemicarbazone) derivatives with reduced toxicity and similar biological activity. Co-administration of two thiosemicarbazone derivatives (ATSM and GTSM) along with copper, namely Cu-GTSM and Cu-ATSM (Cu prototypes), have various cell metabolism and reduction potential and have selectively targeted prostate cancer cells [32]. Co-administration with Cu makes them more active than the corresponding ligand, and an increase in intracellular Cu concentration is associated with their activity. Cu-ATSM is effective at higher doses than Cu-GTSM against different cancer cells. Cu-GTSM also mediated its anti-proliferative potential via increased ROS generation, cathepsin release, lysosomal membrane permeabilization, and apoptotic cascades [33] (Table 1).

Cu ionophores represent a promising therapeutic approach for managing metal dyshomeostasis diseases [42,43]. Long-term usage of ionophores might perturb the usual physiological roles of essential metalloproteins by affecting the homeostasis of biometals. Increased copper levels in cancerous tissues and ionophore administration make cancerous cells more susceptible to ionophore-copper toxicity than normal cells. However, a higher therapeutic selectivity (normal versus cancerous cells) is needed to elucidate a potent drug. Conjugating ionophores with targeting moieties might reduce metal-binding agents’ toxicities and further increase their efficacies [44]. Protecting the copper-binding site might also avoid unwanted systemic chelation. These targets can be easily achieved by specific activation of metal ionophores at their site of action.

Proinophores (site-specific activation of prodrugs) can be achieved by utilizing numerous specific intracellular enzymes, which could further emphasize the urge to target elective enzymes overexpressed in cancerous cells. These enzymes must fully hydrolyze proionophores in cancerous cells to exhibit maximum anti-cancerous potential [45]. The glucoconjugation strategy has been widely used to develop proionophores since they possess numerous benefits, including water solubility, limiting side effects, and enhanced targeting [46]. Galactose, glucose, and moieties have been used with hydroxyquinolines (HQ) molecules to obtain proionophores [47].

## 3. Calcium, Zinc and Potassium Ionophores in Cancer

Ca^2+^ ion contributes significantly to numerous cell signaling pathways that further control a more comprehensive range of cellular processes via interacting with various signaling cascades. Calcium ions are essential in cytosolic signaling as second messengers, and thus cytosolic Ca^2+^ (Ca^2+i^) concentration remains a significant regulator of vital cellular phenomena, including cell proliferation and apoptosis [48]. Thus, intracellular Ca^2+^ concentration should always be adequately maintained by calcium-permeable channels such as store-operated channels (SOCs), transient receptor potential (TRP) channels, mitochondrial calcium uniporter (MCU), and others for normal cell functioning. Ca^2+^/calcineurin and Ca^2+^/calmodulin have emerged as significant checkpoints during cell cycle progression. Ca^2+^-dependent metastasis processes include cell deformation, migration, invasion, and adhesion [49,50]. TRPM7 (transient receptor potential ion channel subfamily M, member 7) channels have been involved in the formation of transient and local calcium domains (calcium flickers) at lamellipodia which guides migration direction [51,52]. Ca^2+^ transport-mediated growth and metastasis are reported in prostate, lung, and breast carcinomas [52]. It has also been observed that apoptotic cells augment intrinsic Ca^2+^ either by releasing calcium from stressed ER or by continual Ca^2+^ influx through activated channels. Cancerous cells display higher apoptotic resistance by calcium influx inhibition either via down-regulating channels or adjusting chronic-reduced ER Ca^2+^ [53].

### 3.1. Zinc Ionophores in Cancer

Research has displayed a strong association between zinc and cancer development. Different malignancies are known to develop and progress in response to zinc imbalance. Evidence also suggests that disruption of FCZ homeostasis occurs early in cancer development. Reduced zinc levels are generally detected in neoplastic tissue, and zinc transporter expression may also play a role in carcinogenesis. It is interesting to note that prostate cancer has been treated with higher levels of cellular zinc or zinc plus cadmium. Remember that the ^2+^ oxidation state of cadmium is stabilized by the presence of a core of filled subshells. Zinc ionophores have been used to increase zinc transport across plasma membranes [Zn^2+^]. Notably, compared to more traditional medicines, zinc-based cancer therapy methods seem to have substantially lower levels of cytotoxicity. Zinc cations interfere with anaphase-A chromosome movement and mitosis through negative shielding charges bounded at microtubule and centrosomes. Cell responses to Zn^2+^ are highly significant for chemotherapeutics. Zinc dysregulation has been implicated in the development and progression of various carcinomas, including deregulation of free cytosolic zinc concentration (FCZ) early homeostasis in cancer development. Zinc transporter expression has also been reported in carcinogenesis, while reduced zinc levels were reported in neoplastic tissue. Increased cellular zinc or zinc+cadmium has shown significant potential in prostate cancer treatment with minimal cytotoxicity. Zn^2+^ concentration is augmented by utilizing zinc ionophores to facilitate zinc transport across the plasma membrane [54]. For instance, Clioquinol (zinc ionophore) exhibited potential anticancer activity. Chloroquine (antimalarial agent), along with zinc, has also shown significant efficacy in clinical trials of human ovarian cancer cells (A2780) via inducing apoptosis [55].

### 3.2. Potassium Ionophores in Cancer

Programmed cell death may be affected by changes in the transmembrane gradients of some physiological ions. According to recent studies, apoptosis may be promoted or inhibited by intracellular calcium increases depending on their level, timing, and location. In contrast, intracellular potassium loss is thought to promote apoptosis. Strong evidence currently suggests that excessive potassium (K^+^) efflux and intracellular K^+^ depletion are critical early steps in apoptosis, in contrast to the ionic process of necrosis, which involves Ca^2+^ influx and intracellular Ca^2+^ buildup. Apoptotic effectors are suppressed by intracellular K^+^ concentration at physiological levels. Activating crucial steps in the apoptotic cascade, such as caspase cleavage, cytochrome c release, and endonuclease activation, a significant loss of cellular K^+^, which is probably a common occurrence in the apoptosis of many cell types, may act as a disaster signal that permits the execution of the suicide program. K^+^ homeostasis disturbance that promotes apoptosis can be caused by excessively active K^+^ channels or ionotropic glutamate receptor channels and is most often accompanied by decreased K^+^ uptake because of Na^+^, K^+^-ATPase failure. Four ionophores were created for potassium: hemispheres, mono- and biscrown ethers (like potassium ionophore III), and antibiotics. Due to its excellent selectivity and widespread use in clinical analyzers, valinomycin is the most used K^+^ ionophore. Because they are inexpensive, mono crown ether sensors frequently exhibit a modest level of selectivity enhancement. Biscrown ethers, which are also capable of forming intramolecular sandwich complexes and interacting with the internal cavity of the crown ether ring, produce noticeably superior results. From this review, it can be concluded that maintaining zinc, calcium, copper, and potassium homeostasis is essential for maintaining human health and immunity.

## 4. Natural Polyether Ionophores Antibiotics as Potent Anticancer Agents

**Figure 1:**
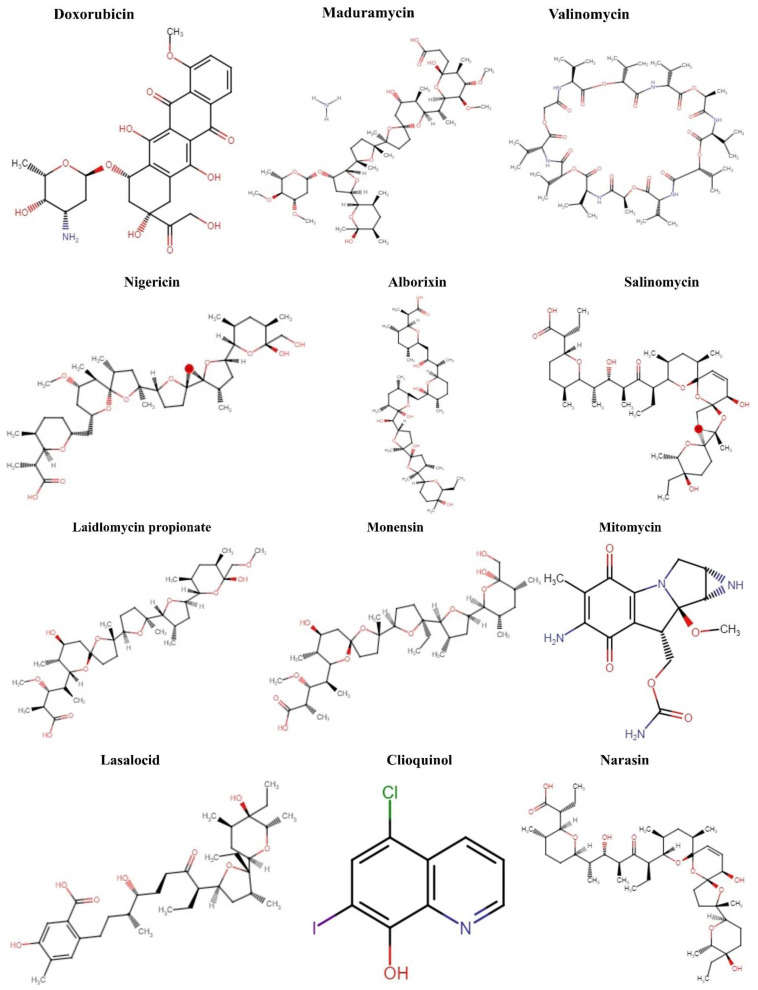
Chemical structure of metallic and polyether ionophores.

Natural polyether ionophores are potent antibiotics that belong to the families of naturally occurring ionophores. It has been evident that ionophores refer to the binding of a molecule to a metal ion that facilitates its transportation via the cell membrane. These chemical and physiological properties of polyether ionophores made it a valuable tool for studying the mechanisms associated with cation transport. Polyether ionophores (squalene derivatives) exhibit a wider range of higher biological activities. Polyether ionophores are highly significant and sizable classes of naturally occurring chemicals. In recent years, there has been an increase in interest in this kind of chemical. Over 120 known naturally occurring ionophores have been identified. Controlling coccidiosis is the primary commercial use of ionophores. They are also applied to ruminants to encourage growth. These substances increase production efficiency by precisely targeting the ruminal bacterial population. Ionophores were the antimicrobials most frequently employed in beef cattle production in 2003. The ionophores with the highest combined annual sales were lasalocid (C_34_H_54_O_8_) (Avatec, Bovatec, Australia), monensin (C_36_H_62_O_11_) (Coban, Rumensin, and Coxidin, United States), and salinomycin (C_42_H_70_O_11_) (Bio-cox, Sacox), narasin (C_43_H_72_O_11_) (Monteban, Maxiban), maduramycin (C_47_H_83_NO_17_) (Cygro, United States), and laidlomycin propionate (C_40_H_66_O_13_) (Cattlyst, United States). Ion-selective electrodes can be made using ionophores as well (Figure 1).

Phycotoxins (marine polyether ionophores) are primarily produced by phytoplankton, fungi, diatoms, cyanobacteria, dinoflagellates, bacteria, and a few marine invertebrates [56,57]. Anticancer antibiotics can inhibit the growth progression of cancer cells in every stage of the cell proliferation cycle (at G0, S, M/G2 phase) by inducing cell cycle arrest, such as cyclin non-specific drugs. In contrast, other antibiotics could induce apoptosis by targeting specific apoptotic genes such as Bcl-2/Bax, caspase-3/8/9, and p53 in cancer patients. These antibiotics further could be exploited as anti-metastasis or EMT regulatory agents for the inhibition of metastasis of cancer cells, such as ciprofloxacin (C_17_H_18_FN_3_O_3_) (pro-apoptosis role), salinomycin (growth inhibition), and nigericin [58,59,60]. Numerous research has speculated that cancer patients seem more sensitive to these emerging anticancer antibiotics that have not been previously exposed. Anticancer antibiotics have been increasingly essential, from the first anticancer antibiotics such as doxorubicin (C_27_H_29_NO_11_), epirubicin (C_27_H_29_NO_11_), and mitomycin (C_15_H_18_N_4_O_5_) to the anticancer antibiotics adriamycin, salinomycin, and fluoroquinolones discovered in recent years. Thus, we have summarized two major antibiotics that have been recently repurposed for cancer treatment, such as nigericin and salinomycin, in subsequent sections.

### 4.1. Nigericin as Potent Therapeutic Candidate for Cancer Management

**Figure 2:**
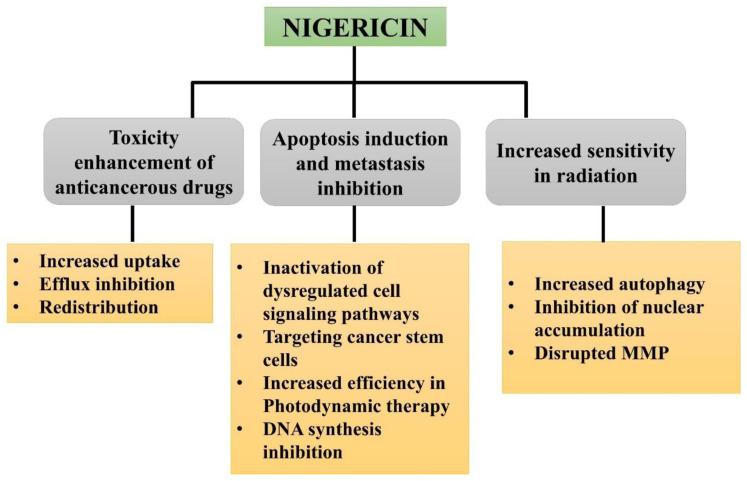
Anti-cancerous potential of nigericin with their associated mechanism.

Nigericin (antibiotic) derived from *Streptomyces peucetius* has been recognized as a potent polyether ionophore used mainly in veterinary medicine. Nigericin has exhibited promising anticancerous potential in several carcinomas, including prostate cancer, colorectal cancer, ovarian cancer, bladder carcinoma, leukemia, pancreatic cancer, and breast carcinoma. Figure 2 displays the efficacy of nigericin against anticancerous drugs via reducing pH and increasing the toxicity of anticancerous drugs. Extracellular pH of tumor is generally lower (about 0.5 pH units) than normal tissues, and cells might survive in these acidic conditions due to the antiports in their membrane exchanges of HCO^3−^ for Cl^−^ and Na^+^ for H^+^ that helps in regulating the intracellular pH. Later, DNA synthesis inhibition was reported in nigericin-treated cancer cells without significant reduction in MMP or ATP concentration [61,62]. Further research reported nigericin-induced alterations in intracellular ion concentrations in neurons, synaptosomes, and C6 glioma cells.

Recent research reported loss of K+ and intracellular acidification, increased lactate production, oxygen consumption, and reduced cellular energy level in nigericin-treated cancer cells [63]. Nigericin (K+ ionophore) exhibited significant inhibitory potential against several essential target proteins of Wnt signaling pathways such as Wnt5a/b, LRP6, and β-catenin. Research further reported significant nigericin-induced cell growth inhibition during prolonged serum starvation, normal culture conditions, and lung tumorspheres [64,65]. Wang et al. reported the dose-dependent nigericin induced inhibited invasion and migration of epithelial ovarian cells via suppressing the EMT transition.

In 1998, Zanke et al. reported nigericin-mediated intracellular acidification leading to cell death induction by activating JNK or stress-activated protein kinases (SAPK) signaling pathways and Bax expression in human MGH-U1 (bladder cancer) cells [66]. Later, nigericin showed remarkable inhibitory potential in colorectal cancers by directly targeting the critical β-catenin destruction complex. Nigericin was also identified as a potent gp170 inhibitor (cancer stem cells inhibitor) in MDR cancer cells and was also reported to kill selective cancer stem cells and overcome multidrug resistance in malignant tumors [67]. In nasopharyngeal cancer, nigericin was also reported to target cancer stem cells and sensitize them to cisplatin drugs in both in vivo and in vitro studies [68]. Although nigericin exhibited promising anticancerous efficacies in several malignancies, its adverse effects cannot be ignored. Thus, it is imperative to develop other drug administration systems for increased bioavailability, avoidance of adverse effects, and improved therapeutic potential.

### 4.2. Salinomycin as Potent Therapeutic Candidate for Cancer Management

Salinomycin is a natural polyether antibiotic isolated from the *Streptomyces albus* strain. Gupta et al. (2009) reported salinomycin as a potent anticancer drug for the first time while screening approximately 16,000 compounds for their anti-cancerous potential against cancer stem cells [69,70]. They reported salinomycin as a more effective anticancer drug than paclitaxel drug. Later, other research studies have also projected its selectivity in targeting cancer stem cells in various cancer cells. Functionally, SAL is one of the membrane ionophores that exhibits a strong affinity for alkali cations, particularly for K. Mounting evidence implicates SAL’s ion transport capabilities as the cause of its phenotypic effects. Apoptosis in human lymphoma cells was previously thought to be induced by a drop in intracellular K concentration. Potassium channels of the mitochondrial and cytoplasmic membranes, which are overexpressed in many human cancer cells, play crucial roles in the regulation of tumorigenesis, tumor cell proliferation, cell cycle progression, and apoptosis and may represent novel and promising molecular targets for cancer therapy.

**Figure 3:**
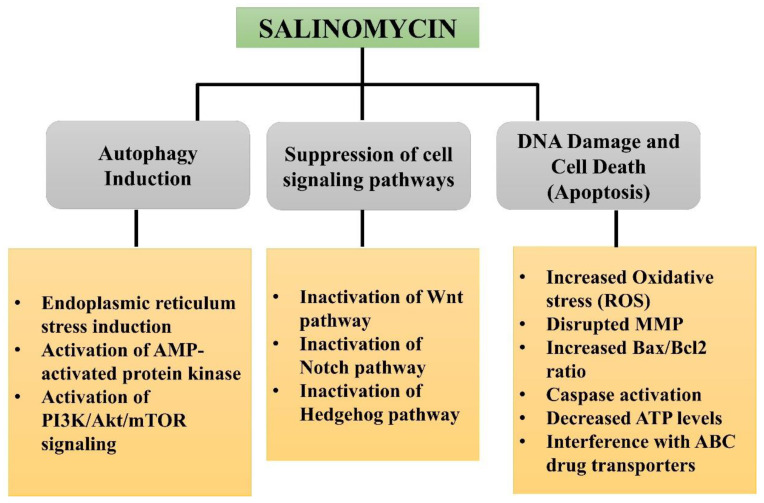
Anti-cancerous potential of salinomycin with their associated mechanism.

Salinomycin exhibited these anti-cancerous efficacies via targeting multiple cell signaling pathways (Figure 3). It was shown that SAL treatment inhibits the Wnt signaling pathway, which is implicated in cancer and embryogenesis, by many mechanisms; other pathways that were blocked by SAL treatment include K-Ras (Kirsten rat sarcoma viral oncogene homolog) and regulation of Hedgehog signaling. It has been demonstrated that changes in genes associated with stemness-related pathways, such as Wnt, Notch, and Hedgehog, are crucial for the development of breast cancer [71,72,73,74]. Salinomycin treatment showed a significant increase in cell death in cancer stem cells and numerous cancer cells through apoptosis induction, enhanced oxidative stress, and autophagy inhibition [75,76]. Salinomycin modulated cancer metastasis via inhibition of cancer cell migration and invasion by targeting EMT (epithelial-mesenchymal transition), Hedgehog signaling and Wnt cell signaling pathways in several cancers, including breast cancer [77]. Salinomycin has shown significant potential in sensitizing several cancers and enhancing the efficacy of commonly used anti-cancer drugs, including doxorubicin, gemcitabine, trastuzumab, and tamoxifen, etc. [78,79,80]. Recent research by Zhang et al. reported increased sensitization to gemcitabine in salinomycin-treated pancreatic cancer by targeting cancer stem cells. Another study reported the sensitizing efficacy of salinomycin to resveratrol in breast cancer [81]. One of the major hindrances in cancer treatment is the resistance of cancerous cells to conventional therapies, and cells escape themselves from eradication. These cells become more aggressive and resistant, leading to cancer relapse.

Most current cancer therapeutic approaches are incompetent in achieving complete cancer elimination and result in cancer relapse after initial remission. Interestingly, salinomycin has also exhibited significant anticancerous potential against numerous multi-drug resistant cancers and developed a drug efflux mechanism by inhibiting several drug transporters such as p-glycoproteins [82]. One major drawback associated with the therapeutic application of salinomycin is toxicity and poor water solubility to normal cells. However, these limitations can get overruled by investigating targeted delivery approaches or synthesizing their more specific and less toxic analogs. Recent research has reported the successful utilization of nanomicelles, nanoparticles, nanotubes, and cell surface markers (CD133 and CD44) conjugated multilamellar liposomes for targeted and efficient delivery of salinomycin [83,84,85,86,87].

Other polyether ionophores, including calcimycin, alborixin, and monexin, have significant anticancerous potential. S100A4 was inhibited by calcimycin in a concentration- and time-dependent manner. Additionally, in cultured keloid fibroblasts (KFs), calcimycin reduced TGF-1-induced expression of collagen type I, fibronectin, smooth muscle actin, and cell viability. Additionally, calcimycin altered the production of Smad7 (SMAD Family Member 7), a TGF-/Smad target gene, and the phosphorylation of Smad2/3, a TGF-1/Smad1-induced gene. The discovery of S100A4 (hypoxia-inducible gene) in KFs was made possible by this study for the first time. S100A4 expression, KF migration, proliferative activity, and extracellular matrix (ECM) production are all inhibited by calcimycin. Together, these findings suggest that calcimycin may be a potential treatment for similar keloid or other fibrotic illnesses [88].

Alborixin exhibited maximum cytotoxic efficacy against human colon carcinoma (HCT-116) cells via elevating intracellular ROS levels accompanied by mitochondrial membrane potential (MMP) loss, reduced Bcl-2 (anti-apoptotic) protein expression level, augmented cleavage of PARP-1 and caspase-3, activated caspase-8, 9 and increased Bax (proapoptotic) protein expression levels [89]. Monesin displayed its proliferation suppression efficacy via inhibiting several growth factor-induced signaling pathways (EGFR). Monesin further reduces the expression level of cyclin D1, cyclin A, and CDK6 and stimulates mitochondria transmembrane potential against numerous human cancer cells [90]. Although oncology has made significant strides in the fight against cancer, people are still waiting for the creation of efficient treatments. Chemotherapy, whose mechanism of action is based on the prevention of cell division, is successful in treating some malignancies but ineffective in treating others. The hunt for novel physiologically active compounds is therefore of utmost significance. Natural substances have been widely employed to treat cancer over the past few decades, and a lot of research suggests that naturally occurring polyether antibiotic is a potential for a brand-new, highly potent anticancer medicine.

## 5. Conclusions

Ion homeostasis disruption is crucial for the survival and proliferation of cancer and comprises a potent target for chemotherapy. Our review has mainly focused on metallic and polyether ionophores’ therapeutic potential, emphasizing their anti-cancerous potential through targeting signaling pathways. Existing drugs, such as antibiotics, have been repositioned as antitumor agents in the recent decade, and the results of more clinical trials on their potency are awaited. Research in this area should target developing novel and effective therapeutics of known pharmaceuticals and exploring new drug delivery systems for managing cancer. Several ionophores, such as metal and polyether ionophores, have displayed potent anticancer potential in several preclinical cancer models, either alone or in combination with other potent anticancer drugs. These medications can be used in antitumor therapy at a lower cost, making treatment more affordable in developing countries. Altogether, the development of target-specific ionophores might provide a novel therapeutic approach for better management of carcinomas.

## Figures and Tables

**Table 1:** Numerous classes of Copper (Cu) ionophores in cancer therapeutics.

Classes of Cu Ionophores	Anticancer Mode of Action	Chemical Structure	Reference
Cu-Pyrrolidine dithiocarbamate (PDTC)	Transport redox-active Cu into the cell Inhibits the cell proliferation, nuclear factor-jB (NF-jB), and ubiquitin–proteasome system inducing apoptosis	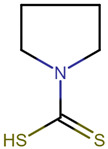	[34]
Cu-DSF (Disulfiram)	Targets stem-like cancer cells and augment the cytotoxic efficacies of several anticancer drugs Inhibited NF-jB up-regulation in ROS-targeted chemotherapeutic approaches. Decreased GSH levels	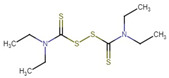	[35,36]
Cu-DPy (disulfide-based Cu carrier, 2,20 -dithiodipyridine)	ROS induction Cytotoxic effects against various cancer cells Increases intracellular Cu concentration Acts as a recyclable Cu ionophore Affect thioredoxin and GSH system	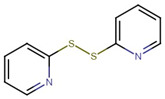	[37]
Cu-GTSM and Cu-ATSM	Possess different reduction potential and cell metabolism Inhibits the chymotrypsin-like activity of the proteasome ROS generation Lysosomal membrane permeabilization cathepsin release Apoptotic cascades	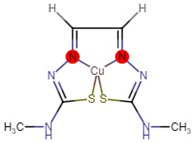	[38]
Cu- Dp44mT (Di-2-pyridylketone-4,4,-dimethyl-3-thiosemicarbazone ) + Dpc (di-2-pyridylketone 4-methyl-4-cyclohexyl-3-thiose micarbazone)	Improved anticancer activity Reduced toxicity Increase in intracellular Cu concentration ROS production Apoptosis induction	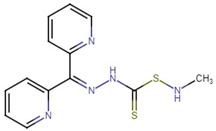	[39]
Elesclomol	Completed a series of clinical trials for patients with advanced melanoma, acute myeloid leukemia, ovarian epithelial cancer, fallopian tube cancer, and primary peritoneal cancer, prostate cancer, and other solid tumors Oxidative stress induction Apoptosis induction Promotes the increase of intracellular Cu levels ROS generation Exert cytotoxicity through DNA-damage Regulates ferroptosis	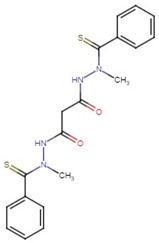	[40,41]
Cu-CQ (7-iodo-5-chloro-8-hydroxyquinoline)	Cell death induction Increased cytotoxicity in combination Increase in intracellular Cu Inhibit the proteasome activity Cytoplasmic XIAP clearance (anti-apoptotic protein) that inhibits caspases activation Apoptosis induction	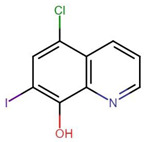	[31]

## Data Availability

Not applicable.
